# Acute Effects of Fast vs. Slow Bench Press Repetitions with Equal Time Under Tension on Velocity, sEMG Activity, and Applied Force in the Bench Press Throw

**DOI:** 10.3390/jfmk10010004

**Published:** 2024-12-26

**Authors:** Athanasios Tsoukos, Michal Wilk, Michal Krzysztofik, Adam Zajac, Gregory C. Bogdanis

**Affiliations:** 1 School of Physical Education and Sport Science, National and Kapodistrian University of Athens, 172 37 Athens, Greece; gbogdanis@phed.uoa.gr; 2 Institute of Sport Sciences, The Jerzy Kukuczka Academy of Physical Education, 40-065 Katowice, Poland; m.wilk@awf.katowice.pl (M.W.); m.krzysztofik@awf.katowice.pl (M.K.); a.zajac@awf.katowice.pl (A.Z.); 3 Department of Sport Games, Faculty of Physical Education and Sport, Charles University in Prague, 110 00 Prague, Czech Republic

**Keywords:** kinematics, kinetics, mean propulsive velocity, accelerometer, linear position transducer

## Abstract

**Background:** The tempo of resistance exercises is known to influence performance outcomes, yet its specific effects on post-activation performance enhancement (PAPE) remain unclear. This study aimed to investigate the effects of fast versus slow repetitions at a load of 70% of one-repetition maximum (1-RM) in the bench press exercise, focusing on velocity, surface electromyographic (sEMG) activity, and applied force while equating time under tension on bench press throw performance. **Methods:** Eleven men (age: 23.5 ± 5.4 years, height: 1.79 ± 0.04 m, body mass: 79.1 ± 6.4 kg, maximum strength 1-RM: 91.0 ± 12.0 kg) participated. Two experimental conditions (FAST and SLOW) and one control (CTRL) were randomly assigned. Participants performed two sets of six repetitions as fast as possible (FAST condition) and two sets of three repetitions at a controlled tempo (SLOW condition) at half the concentric velocity of FAST, as determined in a preliminary session. Before and after the bench press participants performed bench press throws tests (Pre, 45 s, 4, 8, and 12 min after). **Results:** sEMG activity and peak force during the bench press were higher in FAST vs. SLOW conditioning activity (*p* < 0.001), with time under tension showing no significant differences between conditions (*p* > 0.05). Mean propulsive velocity (MPV) during the bench press throw improved equally in both FAST and SLOW conditions compared with baseline from the 4th to the 12th min of recovery (FAST: +6.8 ± 2.9% to +7.2 ± 3.3%, *p* < 0.01, SLOW: +4.0 ± 3.0% to +3.6 ± 4.5%, *p* < 0.01, respectively). Compared to the CTRL, both conditions exhibited improved MPV values from the 4th to 12th min (*p* < 0.01). Peak velocity improvements were observed only after the FAST condition compared to the baseline (*p* < 0.01) with no differences from SLOW. For all muscles involved and time points, sEMG activity during bench press throws was higher than CTRL in both experimental conditions (*p* < 0.01), with no differences between FAST and SLOW. Peak force increased in both FAST and SLOW conditions at all time points (*p* < 0.05), compared to CTRL. **Conclusions:** These findings suggest that post-activation performance enhancement is independent of movement tempo, provided that the resistive load and total time under tension of the conditioning activity are similar. This study provides valuable insights into the complex training method for athletes by demonstrating that varying tempo does not significantly affect post-activation performance enhancement when load and TUT are equated.

## 1. Introduction

During complex training, strength and conditioning coaches and athletes typically pair a heavy load with a lighter load exercise in sequence to enhance force, velocity, and power output [1,2,3,4,5,6,7]. This method was proposed in the early 1970s by Yuri Verkhoshansky [8] and can be described as a comprehensive approach, as it shifts the entire force–velocity curve to the right and upward, improving both strength and speed across the full force–velocity spectrum [1,4,9,10]. Complex training is based on a phenomenon called post-activation performance enhancement (PAPE) [11,12]. It has been shown that the heavy load, known as the conditioning activity, triggers a cascade of various physiological mechanisms that activate the muscles, leading to enhanced performance in the subsequent light load exercise [2,13]. However, the conditioning activity performed with heavy load may also induce fatigue, which suggests that subsequent performance is determined by an interaction between the mechanisms of fatigue and those of PAPE. The factors influencing the interaction between PAPE and fatigue include subjects’ characteristics, such as muscular strength, training level, and muscle fiber type distribution and/or area. Athletes with greater strength [14,15], more experience [16,17], and a higher proportion [18] and/or area of fast-twitch fibers [19] tend to experience greater performance improvements. Additionally, conditioning activity variables, such as volume, intensity, type of muscle contraction, joint angle, muscle length, and velocity loss, also play a role [2,12,13]. Other key variables include the recovery time between the conditioning activity and the subsequent activity [20] as well as the nature of the subsequent activity itself [21]. Regarding the interaction between the volume and intensity of the conditioning activity and recovery time, research has shown that when the conditioning activity intensity exceeds 60% of one-repetition maximum (1-RM) and the number of sets ranges from 1 to 3, the time frame when subsequent performance is enhanced is between 4 and 12 min [3,20,21,22,23,24].

Research has shown that PAPE protocols often lead to increased muscle activation, as measured by surface electromyographic activity (sEMG) [2,3,11,25,26,27]. However, studies examining the acute effects of a conditioning activity on performance, report conflicting findings concerning sEMG activity [25]. Some studies found elevated sEMG activity following a conditioning activity [2,3,25,27,28], while others did not [29,30]. These discrepancies may stem from methodological differences across studies. Generally, sEMG activity is more likely to increase when the exercise involves high-speed, high-force movements, such as ballistic actions, or when muscle fatigue has accumulated [2,3,25,31].

Beyond the factors mentioned above that influence the PAPE phenomenon, an interesting consideration is whether the conditioning activity should be performed at a fast or slow-controlled tempo. Research suggests that faster repetitions increase muscle excitation, as indicated by sEMG [32,33], and also result in greater force and power output [34,35]. Theoretically, faster repetitions during the conditioning activity should activate the involved muscles more effectively, leading to greater improvements in subsequent performance. However, research has also shown that faster repetitions can cause more fatigue when the volume load (i.e., the product of load × sets × repetitions) is kept constant compared to slower repetitions [36], ultimately resulting in performance impairments. Wilk et al. [37] showed that both fast and slow tempos can induce PAPE, but there were differences in its temporal distribution, with peak potentiation occurring at different time points. If a protocol with a constant number of repetitions is used for both tempos, the total time under tension (TUT) will not be equal, or vice versa. Ide et al. [36] investigated the time course of strength and power output after a single bout of strength training performed at both fast and slow velocities. Participants completed five sets of 12 repetition maximum (12-RM) with 50 s of rest between sets and 2 min between exercises (Leg Press at 45° and Leg Extension). They found that maximum strength declined more following the fast repetition protocol compared to the slow protocol (−8.2% vs. −15.1%; *p* < 0.05). However, in that study, the TUT was not equated between conditions [36]. Conversely, another study demonstrated that when TUT is equated, higher loads produce greater fatigue, and when the load is constant, longer TUT leads to more fatigue [37,38]. Due to these inconsistencies, it would be interesting to investigate the interaction between fatigue and PAPE when TUT is equated using the same relative load.

To our knowledge, only one study has investigated the influence of fast versus slow repetitions on subsequent performance. Wilk et al. [39] manipulated the tempo solely during the eccentric phase of the movement (fast 2 s vs. slow 6 s) to assess its effects on power output and velocity during the concentric phase of successive sets of the bench press exercise. The authors concluded that both eccentric tempos improved performance in subsequent sets [39]. However, TUT was not equated in the two conditions, and there was no condition where participants performed the exercise with maximal eccentric velocity.

Given this background and the lack of research examining the acute effects of fast versus slow repetitions with equal TUT on the interaction between PAPE and fatigue, the purpose of the present study was to investigate the influence of fast versus slow repetitions at a load of 70% of 1-RM in the bench press exercise. We selected a 70% 1-RM load for the conditioning activity because evidence suggests that intensities exceeding 60% of 1-RM are effective in eliciting PAPE [3,20,21,22,23,24]. The bench press exercise was selected because it is one of the most widely used upper-body strength exercises in research and athletic training. We noted that no prior research has explored the effects of slow or fast tempos on subsequent power output performance using low-load ballistic exercise. Therefore, to assess the effects of fast or slow bench press exercise with equal TUT as conditioning activity, we used the bench press throw (BPT) with a load of 30% of 1-RM as a subsequent exercise to measure changes in barbell velocity, sEMG activity, and applied force 12 min after the conditioning activity. The importance of the present study lies in the fact that it provides insights into the effects of resistance exercise tempo on acute key performance metrics, which can help inform more effective training strategies (i.e., complex training) for athletes and coaches during different periods of training (preparatory or competitive). By identifying how different tempos influence PAPE, our research offers valuable guidance for optimizing strength and conditioning programs, thereby potentially improving athletic performance and reducing the risk of injury.

## 2. Materials and Methods

### 2.1. Participants

Eleven men with a minimum of 3 years of strength and power training experience, who were involved in recreational individual and team sports (track and field, wrestling, fencing, soccer, and basketball), participated in the study (age: 23.5 ± 5.4 years, height: 1.79 ± 0.04 m, body mass: 79.1 ± 6.4 kg, maximum strength 1-RM bench press: 91.0 ± 12.0 kg, relative strength: 1.2 ± 0.2 kg·kg^−1^). The study was conducted at the start of the transition period. Inclusion criteria for participation were as follows: (a) no use of nutritional supplements or drugs, (b) no musculoskeletal injuries within the past year, (c) non-smoking status, and (d) regular involvement in resistance training for at least 3 years. After a thorough explanation of the testing protocol, possible risks, and the right to withdraw at any time without providing an explanation, written informed consent was obtained from each participant. The study was approved by the local Institutional Review Board (Approval no. 1532/14 June 2023), and all procedures adhered to the Code of Ethics of the World Medical Association (Helsinki Declaration of 1964, revised in 2013).

### 2.2. Research Design

**Figure 1:**
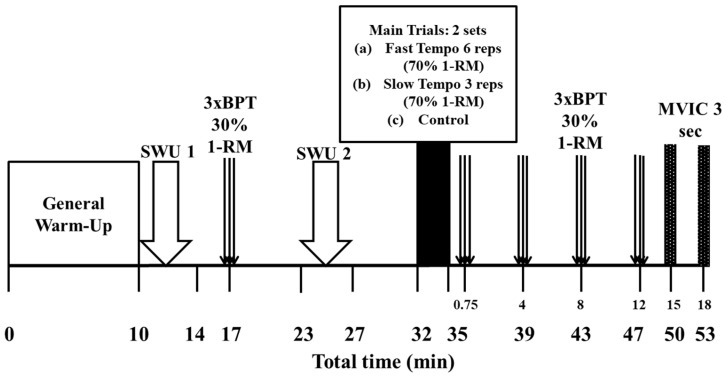
Schematic representation of the experimental protocol. 3xBPT: three repeated bench press throws; SWU: specific warm-up.

The experiment was conducted in the sports performance laboratory of the School of Physical Education at the University of Athens. Measurements were taken at room temperature between 10:00 and 12:00 a.m. This timing ensured consistent environmental conditions and minimized any potential effects from diurnal variations on performance outcomes. A repeated-measures design was employed to investigate the acute effects of bench press exercise on subsequent BPT performance, assessing mean propulsive velocity (MPV), peak velocity (PV), surface electromyographic (sEMG) activity, and peak force (PF). The study included two experimental conditions and one control (CTRL), organized in a randomized and counterbalanced order. A schematic representation of the study design is provided in Figure 1. The participants completed a 10 min general warm-up that included light cycling followed by dynamic stretches. Following the general warm-up, they performed a specific warm-up consisting of two sets of bench press throws at 15% and 30% of their 1-RM. After this, they rested for three minutes. The specific warm-up continued with one set of eight repetitions at 50% of the subsequent load (70% 1-RM), followed by a set of five repetitions at 75% of the load (70% 1-RM), with another 3 min seated rest in between. After a final 5 min rest, participants performed the experimental conditions (FAST or SLOW) or rested (CTRL). They then completed BPTs at four time points into recovery (45 s, 4 min, 8 min, and 12 min). In the first experimental condition, the participants performed two sets of 6 repetitions of the bench press exercise as quickly as possible in both the eccentric and concentric phases of movement, with a load equal to 70% of their 1-RM (FAST condition). In the second condition, participants completed 2 sets of 3 repetitions at a controlled tempo (SLOW condition) which was pre-determined during a preliminary session as 50% of the concentric velocity of the FAST condition. Although the eccentric phase typically exhibits a naturally higher velocity, we controlled the tempo in the SLOW condition by using this lower concentric velocity as a reference for both the eccentric and concentric phases during the conditioning activity. Participants were required not to train for 48 h prior to each lab visit and to maintain the same diet for 24 h prior to each testing session. The dependent variables mentioned below were measured in all conditions in the BPT exercise at the following time points: after warm-up (baseline) and at 0.75, 4, 8, and 12 min. The dependent variables were as follows: (i) mean propulsive velocity (MPV), (ii) peak velocity (PV), (iii) root mean square (RMS) sEMG of the pectoralis major, triceps brachii, and anterior deltoid muscles normalized as a percentage of maximum voluntary isometric contraction (MVIC), and (iv) peak force (PF).

### 2.3. Familiarization and Preliminary Measurements

Participants completed three preliminary sessions. In the first session, they were familiarized with the BPT exercise, and anthropometric data were collected. During the second session, participants’ maximum dynamic bench press strength (1-RM) was measured as described in previous studies [40,41]. Afterward, the participants performed two sets of six repetitions at 70% of their 1-RM load as fast as possible (FAST condition) to measure average concentric and eccentric velocities. Based on this data (mean concentric and eccentric velocities and displacement), the tempo of the second condition (SLOW) was calculated and set to match 50% of the average concentric and eccentric velocities. In the third preliminary session, participants were familiarized with the experimental procedures and completed in randomized order two sets of 6 bench press repetitions at 70% of 1-RM (intending to move as fast as possible) and two sets of 3 repetitions at a slower, individualized tempo, based on the 50% maximum velocity determined in the previous session. The participants were familiarized again with the BPT exercise. The familiarization sessions were conducted during the 10 days preceding the main trial to ensure consistency in technique and readiness for the experimental conditions. Each familiarization session was separated by at least 48 h from the next one.

### 2.4. Maximum Dynamic Strength (1-RM)

Maximum dynamic strength (one-repetition maximum: 1-RM) was assessed during the second preliminary visit on a Smith machine, following the procedures outlined by the National Strength and Conditioning Association (NSCA) [42]. Participants were positioned on the bench, which supported three body segments: the head, shoulders, and hips, with their feet placed flat on the floor, at a knee angle of approximately 90 degrees. A volitional tempo was used to allow the participants to control their movement pace naturally. To ensure safety and provide motivation, two experienced spotters, both experienced strength and conditioning coaches, were present to offer verbal encouragement throughout the assessment.

### 2.5. General and Specific Warm-Up

Participants underwent a standardized warm-up protocol as previously described [3]. Prior to each preliminary or experimental session, they engaged in 5 min of light cycling on a cycle ergometer (at a resistance of 50–60 W), followed by 5 min of dynamic stretching targeting the arm and chest muscles. After the general warm-up, two specific warm-up protocols were performed: the first preceded the baseline measurements of BPTs, while the other was conducted prior to the conditioning activity (Figure 1). The first specific warm-up (SWU 1) consisted of two sets of BPTs: the first set included six repetitions at 15% of 1-RM, followed by a set of four repetitions at 30% of 1-RM. In cases where 15% of 1-RM was less than the weight of the barbell (20 kg), participants performed the SWU-1 with the minimum load available on the Smith machine (i.e., 20 kg). A 3 min rest period in a seated position separated these sets. The second specific warm-up (SWU 2) involved one set of eight repetitions at 50% of the subsequent load (70% 1-RM), followed by a set of five repetitions at 75% of the load that followed (70% 1-RM), with another 3 min seated rest in between.

### 2.6. Main Trials

The two main experimental trials and the control condition (CTRL) were conducted 5–7 days apart in a randomized, counterbalanced order. The conditions were defined as follows:

1. FAST condition (FAST): Participants performed 2 sets of 6 repetitions at 70% of their 1-RM, aiming to move the bar as fast as possible.
2. SLOW condition (SLOW): Participants performed 2 sets of 3 repetitions at 70% of their 1-RM, using a controlled tempo set to 50% of their predetermined maximum concentric and eccentric velocities.
3. Control condition (CTRL): Participants did not perform any conditioning exercise but only completed the BPT.

The participants began all experimental trials with a standardized general and specific warm-up 1 (Figure 1). Following a 3 min recovery period, they performed 3 repeated BPTs at 30% of 1-RM on a Smith machine. The average of the three BPTs was used as the baseline. Six minutes later, participants completed a second specific warm-up, followed by a 5 min rest period. Afterward, they either performed the conditioning exercise (FAST or SLOW) or rested in a seated position for 3 min (CTRL). Following the conditioning exercise, the BPT was re-evaluated at 45 s, and again at 4-, 8-, and 12-min post-conditioning, with participants performing 3 repeated BPTs at each time point.

### 2.7. Bench Press Throws

The BPT exercise was performed on a Smith machine, using a load of 30% of the 1-RM. Participants were instructed to grip the bar as they would in a standard bench press, with their elbows fully extended [2,3]. From this starting position, they executed a rapid countermovement, throwing the barbell as fast and as high as possible for three consecutive attempts without rest in between. Throughout the exercise, participants maintained contact between their heads, torsos, and the bench, with their feet flat on the floor. They were instructed to accelerate the barbell through the full range of motion, lowering it to lightly touch the chest at the sternum before immediately pressing upward to achieve maximum voluntary velocity during the throw. Participants were given the following guidance: “the aim is to lift the bar as fast as possible and lower the barbell fast, but with control” [43].

### 2.8. Movement Velocity Variables and Time Under Tension

Movement velocity was continuously monitored throughout all preliminary and main sessions using a linear position transducer (Tendo Power Analyzer System v. 314, TENDO Sports Machines, Trencin, Slovak Republic). During the bench press exercise on a Smith machine, both mean and peak velocities were monitored for each repetition. In the BPT, the MPV and PV during the concentric phase were obtained. MPV is defined as the average velocity from the start of the concentric phase of the repetition until the moment peak velocity is achieved [44]. For data analysis, we used the average of the MPV and PV from the three BPTs recorded at each time point. We also analyzed the average MV and PV from the first and second sets in each experimental condition. Additionally, we calculated TUT in set 1 and set 2, as well as the total TUT in each experimental condition, from the data obtained from the linear position transducer. TUT was calculated from the data obtained from the linear position encoder and was confirmed by video recordings of the movement using a high-speed camera (Casio Exilim, EX ZR-1000, Tokyo, Japan).

### 2.9. Peak Force Calculations from Acceleration Data

Acceleration was continuously monitored throughout all preliminary and main sessions using a tri-axial accelerometer (PS-3223, Pasco Scientific, Roseville, CA, USA) with a sampling frequency of 100 Hz, placed on the barbell and connected to a laptop via Bluetooth [45]. Peak force (PF) during both the conditioning activity and the BPTs was calculated from the acceleration data using Excel spreadsheets. A fourth-order Butterworth filter with a cutoff frequency of 4 Hz was applied to smooth the accelerometer data [46]. Force was calculated using the following equation: F = (m∙g) + (m∙a), where m is the mass of the barbell, g is the acceleration due to gravity, and a is the measured acceleration. As stated above, for data analysis, we used the average PF from the three BPTs recorded at each time point, as well as the average PF from all the performed repetitions from the first and second sets of each experimental condition.

### 2.10. sEMG Activity

Surface electromyographic (sEMG) activity of the dominant-right pectoralis major (PM-pars sternocostalis), anterior deltoid (AD), and triceps brachii (TB-lateral head) muscles was recorded and analyzed using a Biopac MP35 data acquisition unit and Acqknowledge 4.2.0 software (Biopac Systems Inc., Santa Barbara, CA, USA). Bipolar Ag/AgCl electrodes (interelectrode distance: 20 mm) were applied to the skin following SENIAM guidelines [47] with a ground electrode positioned on the clavicle. To ensure low impedance, the skin was shaved, cleaned with alcohol, and lightly abraded. sEMG signals were recorded at a sampling frequency of 2000 Hz, amplified (gain = 1000), and filtered with a band pass of 30–500 Hz [31,48]. The Root Mean Square (RMS) of each muscle burst was calculated between manually defined onset and endpoints. Peak RMS values were normalized to peak sEMG amplitudes obtained during maximum voluntary isometric contractions (MVICs) in the bench press exercise with elbow angles of 90° and 140°, performed three minutes after the end of each condition. Participants performed two 3-s repetitions of an isometric bench press exercise, with each repetition separated by a 3-min rest interval (Figure 1) [48]. The highest RMS value was used for analysis. All sEMG values were expressed as a percentage of MVIC (%MVIC). We also calculated the average sEMG activity of all muscles.

### 2.11. Statistical Analysis

Statistical analyses were performed using the SPSS Statistics Ver. 23 (IBM Corporation, Armonk, NY, USA). Data are presented as means ± standard deviations (SD). A two-way repeated measures ANOVA was conducted to assess differences across conditions and time points, with a 3 × 5 design for all measured variables (Conditions: FAST, SLOW, CTRL × Time points: Pre, 0.75, 4, 8, 12 min). Additionally, two-way repeated measures ANOVAs (2 Conditions: FAST, SLOW × 2 sets: 1st set, 2nd set) were used to examine differences in the conditioning activity between experimental conditions. When a significant main effect or interaction was found, Tukey’s post hoc test was applied. Effect sizes for main effects and interactions were evaluated using partial eta squared (η^2^p) [49], classified as small (0.01 to 0.059), moderate (0.06 to 0.137), and large (>0.137). For pairwise comparisons, effect size was calculated using Hedges’ g [50], categorized as small (<0.3), medium (0.3–0.8), and large (>0.8). Statistical significance was set at *p* < 0.05.

## 3. Results

### 3.1. Velocity and Time Under Tension During the Conditioning Activity

During the conditioning activity, the average (concentric + eccentric) barbell velocity in the FAST condition across both sets was 0.86 ± 0.12 m s^−1^, compared to 0.42 ± 0.05 m∙s^−1^ (*p* < 0.001) in the SLOW condition, confirming that participants were moving the barbell at approximately half of the speed of the FAST condition.

**Figure 2:**
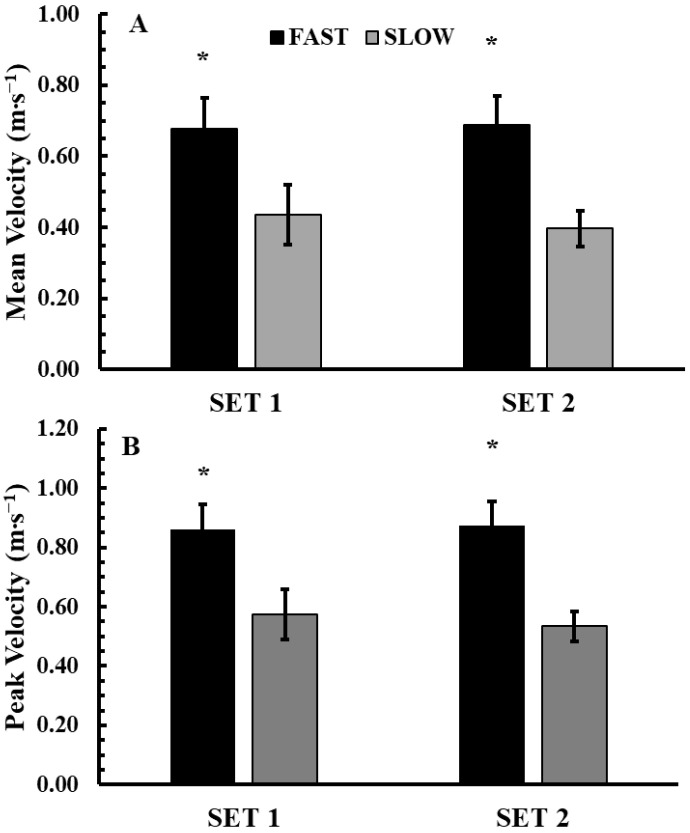
Mean velocity (**A**) and peak velocity (**B**) during the conditioning activity (Fast: 2 sets × 6 repetitions and Slow: 2 sets × 3 repetitions). Mean and peak velocity corresponds to the average values of each set. *: *p* < 0.01 from SLOW.

Mean and peak concentric velocity during the conditioning activity are shown in Figure 2A,B. The 2-way ANOVA (2 conditions × 2 sets) did not show a significant interaction for MV (*p* = 0.087, η^2^p = 0.26). However, there was a significant main effect for condition (*p* < 0.001, η^2^p = 0.94). Tukey’s post hoc test revealed that MV was significantly higher in the FAST condition compared to the SLOW (0.68 ± 0.8 vs. 0.42 ± 0.8 m s^−1^; *p* < 0.001; Hedges’ g = 3.4). The 2-way ANOVA (2 conditions × 2 sets) did not show a significant interaction for PV (*p* = 0.20, η^2^p = 0.16). However, there was a significant main effect for condition (*p* < 0.001, η^2^p = 0.92). Tukey’s post hoc test revealed that PV was significantly higher in the FAST condition compared to the SLOW (0.87 ± 0.10 vs. 0.55 ± 0.11 m s^−1^; *p* < 0.001; Hedges’ g = 2.9).

**Figure 3:**
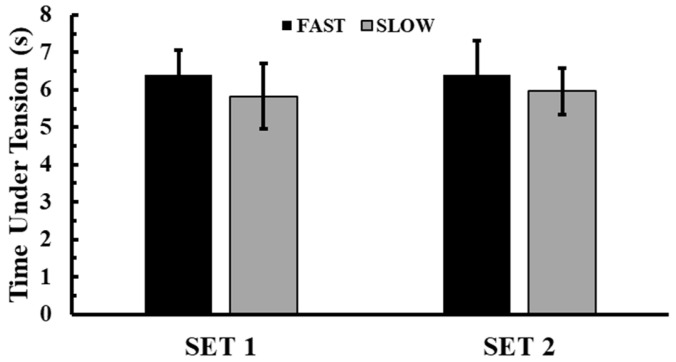
Time under tension during the conditioning activity in the experimental conditions (Fast: 2 sets × 6 repetitions and Slow: 2 sets × 3 repetitions).

Figure 3 illustrates TUT per set in the FAST and SLOW conditioning activity protocols. Analysis via 2-way ANOVA (2 conditions × 2 sets) revealed no significant interaction or main effects (*p* > 0.05).

### 3.2. Time Course of Mean Propulsive Velocity and Peak Velocity During the BPTs

**Figure 4:**
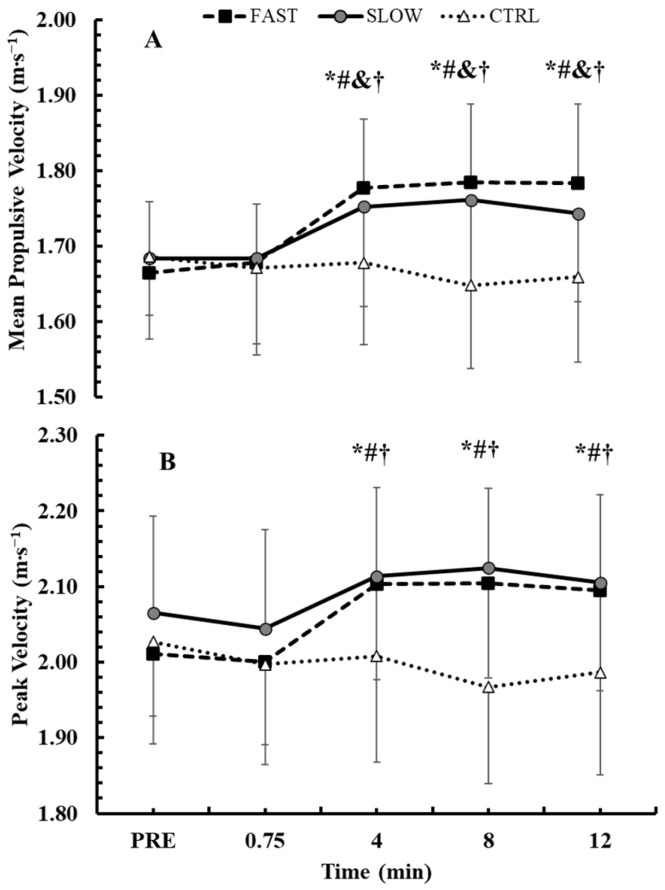
Time course of changes in the BPT mean propulsive velocity (**A**) and peak velocity (**B**). *: *p* < 0.01 from PRE in the FAST condition; &: *p* < 0.01 from PRE in the SLOW condition; #: *p* < 0.01 from CTRL at the corresponding time point in the FAST condition; †: *p* < 0.01 from CTRL at the corresponding time point in the SLOW condition.

Figure 4A presents the time course of MPV performance during the BPTs. Baseline MPV values were consistent across conditions (*p* > 0.95). A 2-way ANOVA (3 conditions × 5 time points) indicated a significant interaction effect between condition and time (*p* < 0.001, η^2^p = 0.61). Tukey’s post hoc tests showed that both experimental conditions led to significant MPV improvements from baseline. Specifically, in the FAST condition, MPV performance improved from the 4th (+6.8 ± 2.9%, *p* < 0.01, Hedges’ g = 1.2) to the 12th min of recovery (+7.2 ± 3.3%, *p* < 0.01, Hedges’ g = 1.2). Similarly, in the SLOW condition, MPV performance increased from the 4th (+4.0 ± 3.0%, *p* < 0.01, Hedges’ g = 0.6) to the 12th min of recovery (+3.6 ± 4.5%, *p* < 0.01, Hedges’ g = 0.5). Compared to the CTRL condition, both FAST and SLOW conditions yielded significantly greater MPV values from the 4th to the 12th min of recovery (FAST: *p* < 0.01, Hedges’ g = 1.7 to 1.8; SLOW: *p* < 0.01, Hedges’ g = 1.4 to 1.5). No differences were observed between the FAST and SLOW conditions.

The time course of PV during the BPTs is presented in Figure 4B. Baseline PV values were similar across conditions (*p* > 0.18). A 2-way ANOVA (3 conditions × 5 time points) identified a significant interaction effect between condition and time (*p* < 0.001, η^2^p = 0.43). Tukey’s post hoc tests showed that PV performance improved from baseline only in the FAST condition. Specifically, in the FAST condition, PV performance increased from the 4th (+4.7 ± 5.5%, *p* < 0.01, Hedges’ g = 0.7) to the 12th min of recovery (+4.3 ± 5.0%, *p* < 0.01, Hedges’ g = 0.6). Although PV performance did not significantly improve in the SLOW condition compared with its respective baseline (*p* > 0.05), there was an increase with medium effect sizes between the 4th and 12th mins of recovery (Hedges’ g = 0.40 to 0.50). Compared to the CTRL condition, both FAST and SLOW conditions resulted in greater PVs from the 4th to the 12th min of recovery (FAST: *p* < 0.01, Hedges’ g = 0.7 to 1; SLOW: *p* < 0.01, Hedges’ g = 0.8 to 1.2). No significant differences were observed between the FAST and SLOW conditions.

### 3.3. sEMG Activity During the Conditioning Activity

**Figure 5:**
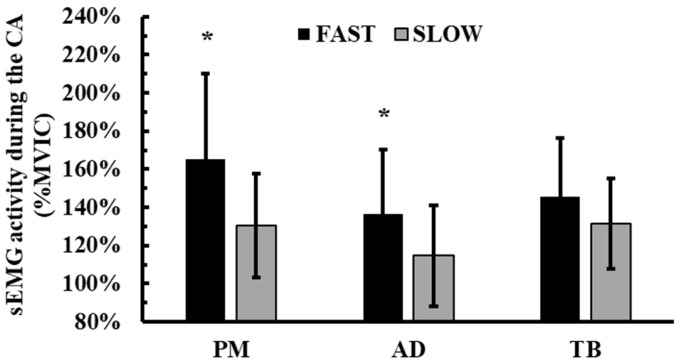
Changes in sEMG activity during the conditioning activity across the experimental conditions. *: *p* < 0.01 from SLOW. PM: pectoralis major muscle; AD: anterior deltoid muscle; TB: triceps brachii muscle.

The 2-way ANOVA (2 conditions × 2 sets) did not show a significant condition × time interaction for sEMG activity of PM (*p* = 0.97, η^2^p = 0.0001), sEMG of TB (*p* = 0.75, η^2^p = 0.01), sEMG of AD (*p* = 0.61, η^2^p = 0.03), and the average sEMG of all muscles (*p* = 0.78, η^2^p = 0.008). However, there was a significant main effect of condition for PM (*p* = 0.01, η^2^p = 0.49), AD (*p* = 0.02, η^2^p = 0.42), and the average of all muscles (*p* = 0.01, η^2^p = 0.50). Tukey’s post hoc tests showed that sEMG of PM muscle (FAST: 165.5 ± 44.4% vs. SLOW: 130.4 ± 27.1%; *p* = 0.01, Hedges’ g = 0.9), sEMG AD (FAST: 136.7 ± 33.4% vs. SLOW: 114.6 ± 26.6%; *p* = 0.02, Hedges’ g = 0.7), and the average sEMG of all muscles (FAST: 146.9 ± 26.9% vs. SLOW: 125.5 ± 19.1%; *p* = 0.01, Hedges’ g = 0.9) was greater during the FAST condition compared to CTRL (Figure 5).

### 3.4. Time Course of sEMG Activity During the BPTs

**Figure 6:**
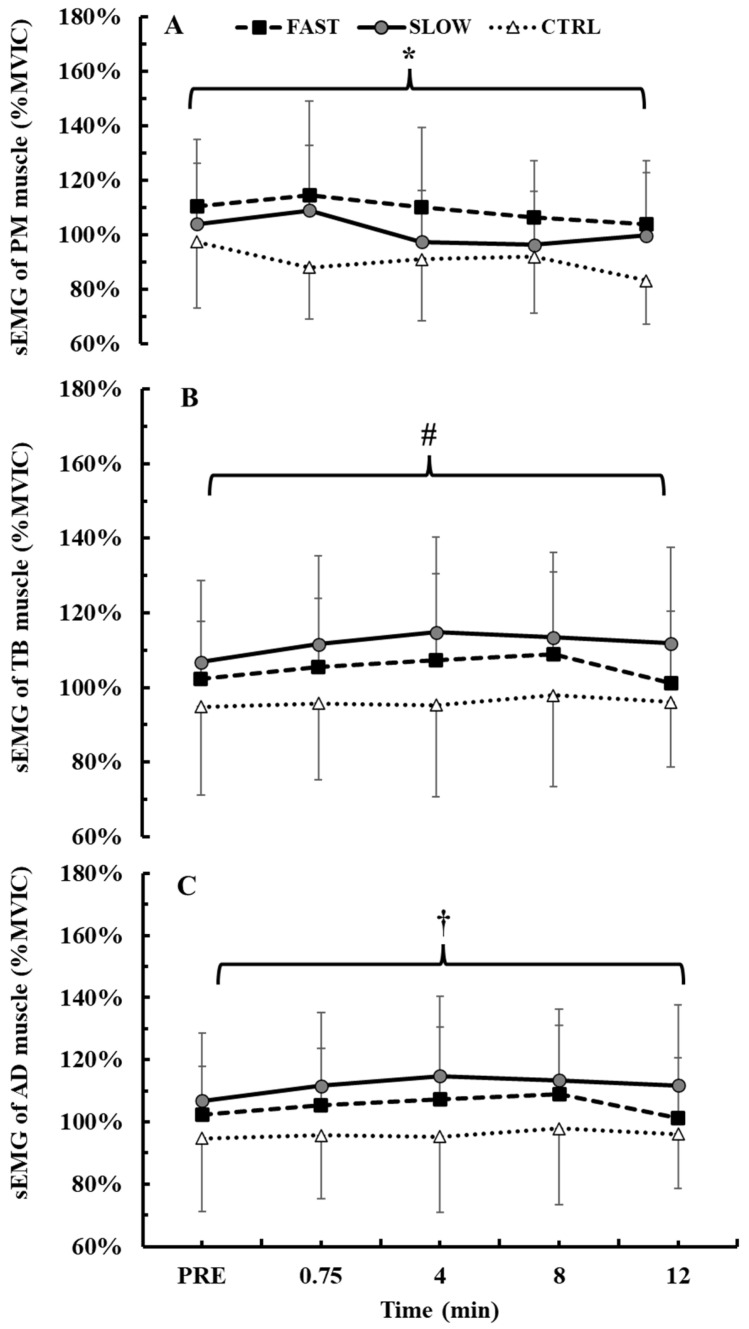
Surface electromyographic activity (sEMG) activity during the bench press throws across the conditions. PM: pectoralis major (**A**); TB: triceps brachii (**B**); AD: anterior deltoid (**C**). Main effect condition. *: *p* < 0.01 FAST from CTRL; #: *p* < 0.01 FAST and SLOW from CTRL; †: *p* < 0.01 SLOW from CTRL.

The 2-way ANOVA (3 conditions × 5 time points) did not show a significant condition × time interaction for sEMG activity of PM muscle (*p* = 0.07, η^2^p = 0.16). However, there was a significant main effect for condition (*p* < 0.001, η^2^p = 0.43). Tukey’s post hoc tests showed that sEMG of PM muscle was greater during the FAST condition compared to CTRL (109.1 ± 26.2% vs. 90.4 ± 20.5%, *p* < 0.01, Hedges’ g = 0.8). No difference was observed between FAST and SLOW conditions (*p* = 0.27) (Figure 6A). The 2-way ANOVA (3 conditions × 5 time points) did not show a significant condition × time interaction for sEMG activity of TB muscle (*p* = 0.43, η^2^p = 0.09). However, there was a significant main effect for condition (*p* < 0.001, η^2^p = 0.53) and for time (*p* = 0.001, η^2^p = 0.28). Tukey’s post hoc tests showed that sEMG of TB muscle was greater during the FAST and SLOW conditions compared to CTRL (FAST: 113.9 ± 17.5%, SLOW: 118.5 ± 26.4% vs. CTRL: 88.4 ± 15.0%, *p* < 0.01, Hedges’ g = 1.5 and 1.4). No difference was observed between the FAST and SLOW conditions (*p* = 0.78) (Figure 6B). Regarding the main effect for time, Tukey’s post hoc tests showed that sEMG of TB muscle was lower at the 12th min of recovery in all conditions (*p* < 0.01).

The 2-way ANOVA (3 conditions × 5 time points) did not show a significant condition × time interaction for sEMG activity of AD muscle (*p* = 0.79, η^2^p = 0.06). However, there was a significant main effect for condition (*p* < 0.05, η^2^p = 0.38). Tukey’s post hoc tests showed that sEMG of AD muscle was greater during the SLOW condition compared to CTRL (111.7 ± 23.2% vs. 95.9 ± 21.4%, *p* = 0.01, Hedges’ g = 0.7) (Figure 6C). No difference was observed between the FAST and SLOW conditions (*p* = 0.51).

The 2-way ANOVA (3 conditions × 5 time points) did not show a significant condition × time interaction for sEMG activity of the average of all muscles (*p* = 0.40, η^2^p = 0.10). However, there was a significant main effect for condition (*p* < 0.001, η^2^p = 0.72) and for time (*p* = 0.01, η^2^p = 0.33). Tukey’s post hoc tests showed that sEMG of all muscles was greater during the FAST and SLOW conditions compared to CTRL (FAST: 109.3 ± 11.9%, SLOW: 110.5 ± 15.6% vs. CTRL: 91.6 ± 11.4%, *p* < 0.01, Hedges’ g = 1.5 and 1.3). No difference was observed between the FAST and SLOW conditions (*p* = 0.92). Regarding the main effect for time, Tukey’s post hoc tests showed that sEMG of all muscles was lower in the 12th min of recovery in all conditions simultaneously compared to all time points (*p* < 0.01).

### 3.5. Peak Force of the Conditioning Activity

**Figure 7:**
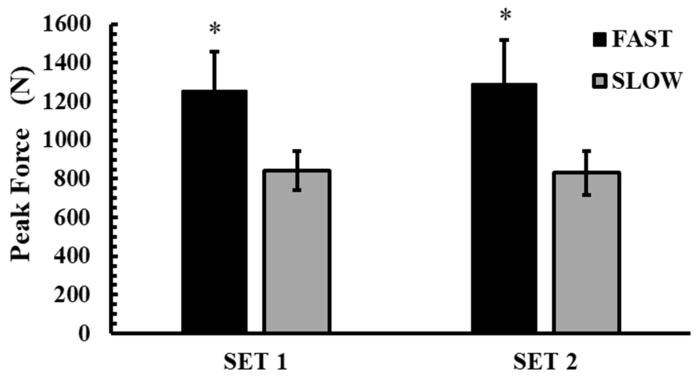
Peak force during the conditioning activity across the experimental conditions. *: *p* < 0.01 from SLOW to FAST.

Peak force during the conditioning activity is shown in Figure 7. The 2-way ANOVA (2 conditions × 2 sets) showed a significant interaction for PF (*p* = 0.037, η^2^p = 0.37). Tukey’s post hoc tests revealed that PF was significantly higher in the FAST condition compared to the SLOW in both sets (1st SET: 1257.0 ± 203.1 N vs. 840.3 ± 100.7 N, *p* < 0.001; Hedges’ g = 2.5; 2nd SET: 1293.2 ± 226.7 N vs. 830.8 ± 112.6 N, *p* < 0.001; Hedges’ g = 2.6).

### 3.6. Time Course of Peak Force During the BPTs

**Figure 8:**
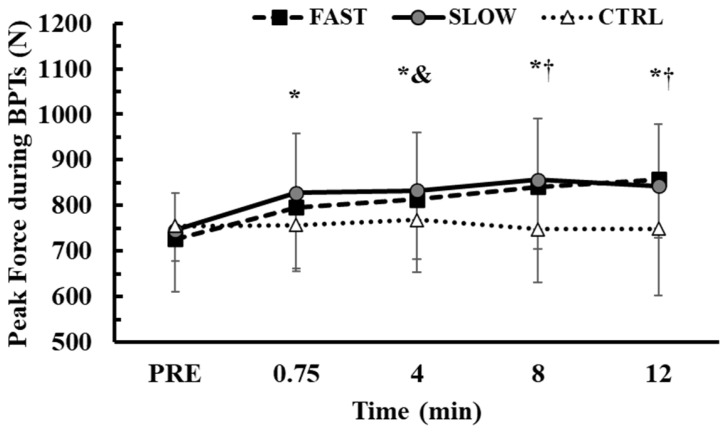
Time course of peak force in the bench press throws across the experimental conditions. *: *p* < 0.05 from PRE in the FAST and SLOW conditions; &: *p* < 0.01 from CTRL at the corresponding time point in the SLOW condition; †: *p* < 0.01 from CTRL at the corresponding time point in the FAST and SLOW conditions.

The time course of PF performance changes during the BPTs is presented in Figure 8. Baseline PF values were similar across conditions (*p* > 0.98). A 2-way ANOVA (3 conditions × 5 time points) identified a significant interaction effect between condition and time (*p* < 0.001, η^2^p = 0.32). Tukey’s post hoc tests showed that PF increased from baseline in FAST and SLOW conditions from 0.75 min (FAST: +9.6 ± 9.0%, *p* < 0.05, Hedges’ g = 0.5; SLOW: +10.8 ± 11.6%, *p* < 0.01, Hedges’ g = 0.7) to the 12th min of recovery (FAST: +18.5 ± 11.1%, *p* < 0.01, Hedges’ g = 1.02; SLOW: +12.7 ± 9.8%, *p* < 0.01, Hedges’ g = 0.8). Compared to the CTRL condition, PF was higher during the FAST and SLOW conditions at the 8th and 12th mins of recovery (*p* < 0.01; Figure 8).

## 4. Discussion

The main finding of the present study was that both FAST and SLOW conditioning activities with equal TUT led to a significant potentiation of the BPTs. MPV and PV were higher from baseline and CTRL from the 4th to the 12th min of recovery in both conditions. Also, PF improved equally in both FAST and SLOW conditions from the 4th min onwards. These improvements in performance were accompanied by elevated sEMG activity in all muscles evaluated. These findings collectively indicate a similar potentiation of neuromuscular function during BPTs following both FAST and SLOW conditioning activities with equal TUT.

This is the first study comparing the effects of FAST versus SLOW conditioning activities on BPT performance using the same load (70% 1-RM) and equal TUT. We hypothesized that the SLOW condition would yield lower and earlier potentiation effects than the FAST, as previous research indicates that fast repetitions generate greater neuromuscular activation [33] and fatigue [36] compared to SLOW. However, our findings showed equal PAPE in both conditions, probably due to the matched TUT and load (70% of 1-RM). Tran et al. [37] demonstrated that fatigue varied with TUT and load, with greater fatigue occurring in slower repetitions when load was equated or in heavier loads when TUT was equated. This supports our finding that with equal TUT and load, neuromuscular responses are comparable.

The potentiation of BPT performance was observed between the 4th and the 12th min of recovery in both conditions, in accordance with previous research [2,3,20,22] and its time course was strikingly similar. Studies indicate that when the intensity of the conditioning activity is between 60% and 80% of 1-RM and the set count ranges from 1 to 3, the optimal recovery time for enhancing BPT performance falls within this 4–12 min window, provided that the number of repetitions is kept below seven [2,3,24]. If the number of repetitions is more than that, then the potentiation of MPV is delayed, and 10–12 min of recovery may be required [2,3]. The current study aligns with these findings, confirming the benefits of performing only 3–6 repetitions to enable substantial performance potentiation irrespective of movement tempo, provided that TUT is equated.

Another finding of the present study was that sEMG activity was higher during the BPTs after FAST and SLOW protocols compared to CTRL. Research has shown that after a conditioning exercise sEMG activity of prime movers increases [2,3,25,27,28] in parallel with performance. This result may be due to an increase in neural drive to the working muscles, which would result in a large number of higher-order motor units being activated with a higher firing rate [11]. We also found that PF in the BPTs significantly increased (Figure 7). As sEMG activity is highly related to muscle force and rate of force development [31,51], it is expected to have high and equal sEMG activities during the BPTs performed after both FAST and SLOW conditioning activities [31,51,52].

Equal increases in performance and sEMG activity during the BPTs were observed despite the fact that during the conditioning activity movement velocity and sEMG were higher in the FAST condition. This may indicate that increased sEMG during the conditioning activity is not the only mechanism causing performance enhancement. Other mechanisms such as increased muscle temperature [11] may also contribute to BPT improvements, more so in the SLOW condition. Resistance exercise has been shown to elevate both muscle and skin temperatures [53,54], and muscle temperature positively correlates with power performance [55,56]. Increased muscle temperatures are linked with increased sEMG amplitude [57], higher firing rates [58], enhanced anaerobic ATP turnover rates, and contractile efficiency of muscle fibers [59]. Therefore, the enhanced BPT performance in the SLOW condition may be partially attributed to muscle temperature, even though sEMG during the conditioning activity was lower. Unfortunately, muscle temperature was not measured in the present study, and this is a limitation for providing a physiological explanation for the equal effects of FAST and SLOW conditioning activities on subsequent BPTs. An additional limitation of the present study was the relatively small sample size (n = 11). While the strict inclusion criteria ensured homogeneity among participants, which enhances reliability within this population, the findings may not be generalizable to larger or more diverse populations. Similar sample sizes have been used in previous studies investigating acute neuromuscular responses and biomechanics in trained individuals [3,40,60]. Future studies should consider larger sample sizes to validate and extend these findings. Another potential limitation was that the present study design can be characterized as quasi-experimental. We used a repeated measures design, where each participant acted as their own control, but learning and order effects were possible. To mitigate such effects, we implemented randomization of the sequence in which the conditions were performed, ensuring the validity of the comparisons.

Strength and conditioning coaches can apply these findings by selecting the tempo of the conditioning activity based on the training goals. A faster tempo may be more suitable when the primary objective is to enhance muscle power [61], such as late in the competition period [62]. In contrast, a slower tempo may be more appropriate when the aim is to develop muscle power along with motor control and technique, such as during the preparatory phase of training. Slower tempos have been shown to challenge velocity consistency more and may require greater attentional focus, which is crucial for enhancing motor control and coordination [63]. This suggests that incorporating slower tempos could be particularly beneficial for training contexts that emphasize precise movement execution and injury prevention.

Future research should focus on the effect of tempo variation on PAPE by exploring additional variables that may influence performance outcomes, such as muscle temperature, muscle pH, muscle blood flow, water content, hormonal responses, and neuromuscular fatigue. It would also be beneficial to examine different resistance training protocols across various sports and populations (e.g., females, older adults). Additionally, exploring longer recovery intervals post-exercise and their effects on PAPE could provide deeper insights into optimizing training regimens for athletic performance. Lastly, integrating biomechanical analyses and advanced imaging techniques could help improve the understanding of the underlying mechanisms driving the effects of different tempos on performance outcomes.

## 5. Conclusions

This study demonstrates that both FAST and SLOW conditioning protocols with an equal TUT significantly enhance neuromuscular performance in BPTs from the 4th to the 12th min of recovery. Although movement velocity, PF, and sEMG were higher during the FAST vs. SLOW conditioning activity, there were no differences in MPV, PV, PF, and sEMG augmentation during the BPTs between the two conditions. Collectively, these findings suggest that PAPE is independent of movement tempo, provided that the resistive load and total TUT of the conditioning activity are similar.

## Data Availability

The data are available upon request from the corresponding author.
